# An open-source tool for converting 3D mesh volumes into synthetic DICOM CT images for medical physics research

**DOI:** 10.1007/s13246-025-01599-x

**Published:** 2025-07-24

**Authors:** Michael John James Douglass

**Affiliations:** 1 School of Physics, Chemistry and Earth Sciences, University of Adelaide, Adelaide, SA Australia; 2 Department of Medical Physics, Royal Adelaide Hospital, Adelaide, SA Australia; 3 Australian Bragg Centre for Proton Therapy and Research, Adelaide, SA Australia; 4 University of South Australia, Allied Health and Human Performance, Adelaide, SA Australia

**Keywords:** Synthetic CT, Medical imaging, Radiation oncology, 3D mesh, DICOM, Treatment planning

## Abstract

Access to medical imaging data is crucial for research, training, and treatment planning in medical imaging and radiation therapy. However, ethical constraints and time-consuming approval processes often limit the availability of such data for research. This study introduces DICOMator, an open-source Blender add-on designed to address this challenge by enabling the creation of synthetic CT datasets from 3D mesh objects. DICOMator aims to provide researchers and medical professionals with a flexible tool for generating customisable and semi-realistic synthetic CT data, including 4D CT datasets from user defined static or animated 3D mesh objects. The add-on leverages Blender’s powerful 3D modelling environment, utilising its mesh manipulation, animation and rendering capabilities to create synthetic data ranging from simple phantoms to accurate anatomical models. DICOMator incorporates various features to simulate common CT imaging artefacts, bridging the gap between 3D modelling and medical imaging. DICOMator voxelises 3D mesh objects, assigns appropriate Hounsfield Unit values, and applies artefact simulations. These simulations include detector noise, metal artefacts and partial volume effects. By incorporating these artefacts, DICOMator produces synthetic CT data that more closely resembles real CT scans. The resulting data is then exported in DICOM format, ensuring compatibility with existing medical imaging workflows and treatment planning systems. To demonstrate DICOMator’s capabilities, three synthetic CT datasets were created: a simple lung phantom to illustrate basic functionality, a more realistic cranial CT scan to demonstrate dose calculations and CT image registration on synthetic data in treatment planning systems. Finally, a thoracic 4D CT scan featuring multiple breathing phases was created to demonstrate the dynamic imaging capabilities and the quantitative accuracy of the synthetic datasets. These examples were chosen to highlight DICOMator’s versatility in generating diverse and complex synthetic CT data suitable for various research and educational purposes, from basic quality assurance to advanced motion management studies. DICOMator offers a promising solution to the limitations of patient CT data availability in medical physics research. By providing a user-friendly interface for creating customisable synthetic datasets from 3D meshes, it has the potential to accelerate research, validate treatment planning tools such as deformable image registration, and enhance educational resources in the field of radiation oncology medical physics. Future developments may include incorporation of other imaging modalities, such as MRI or PET, further expanding its utility in multi-modal imaging research.

**Supplementary Information:**

The online version contains supplementary material available at 10.1007/s13246-025-01599-x.

## Introduction

Advancements in medical imaging and radiation therapy have significantly improved the diagnosis and treatment of various diseases, particularly cancer. Central to these advancements in radiation oncology is the ability to accurately image and simulate patient anatomy and the interaction of radiation with biological tissues. This capability has enabled modern radiation therapy approaches such as volumetric modulated arc therapy (VMAT) and proton therapy.

A significant challenge in medical physics research is the limited access to patient CT data. Such data is valuable for retrospective studies, training machine learning models, and radiomic analyses. Obtaining and using CT data for research typically requires formal approval from hospital ethics committees, a process that can be time-consuming and complex. While open-access datasets are available [1], they may not always provide a suitable patient cohort for specific research projects.

Synthetic data offers an alternative solution in some scenarios, bypassing ethical issues [2] and allowing the creation of more customised datasets tailored to specific research needs. Custom synthetic datasets often provide more controlled environments with fewer uncontrollable variables, allowing for more precise and targeted research.

Synthetic data in radiation oncology has already been extensively utilised for various purposes, including training machine learning models [3–6], validating computational algorithms [7], education [2] and inter-modality medical image conversion [5, 8, 9]. While these applications have contributed to advancing the field of radiation oncology, there has been a limitation on creating customisable, complex, and realistic synthetic datasets that can be seamlessly imported into treatment planning systems.

This limitation has hindered the development of more sophisticated research methodologies and the evaluation of treatment planning algorithms under diverse and challenging scenarios.

To address this gap, we have developed DICOMator [10], a Blender [11] add-on that bridges the robust modelling capabilities of Blender with medical imaging formats such as DICOM (Digital Imaging and Communications in Medicine) [12]. DICOM is the international standard for medical images, ensuring compatibility across different medical devices and software. DICOMator facilitates the voxelisation of 3D mesh objects and their export as DICOM-compliant computed tomography (CT) series, enabling the generation of highly detailed and customisable synthetic CT datasets that closely mimic real patient data, complete with simulated artifacts and anatomical variations. Ultimately, the goal of this tool is to quickly and easily generate synthetic CT images that are similar enough to real CT data for specific use cases.

DICOMator utilises Blender’s [11] native capabilities to process 3D mesh objects, converting them into volumetric data. Blender, an open-source 3D creation software, offers powerful modelling tools, a flexible Python [13] API, and a large community of users and developers, making it an ideal platform for this application. DICOMator optimises the voxelisation process by focusing on relevant areas such as structure boundaries, enhancing overall performance. The tool offers flexible density assignment to structures, allowing users to precisely control material definitions in complex geometries. These features enable researchers to explore a wide range of clinical scenarios without the ethical and logistical constraints associated with real patient data.

Although mesh voxelisation algorithms have been extensively studied [14–16], current solutions predominantly lack intuitive, user-friendly tools tailored for medical physics researchers. Existing workflows typically rely on external three-dimensional models (e.g., Standard Triangle Language [STL] format), followed by manual voxelisation and conversion processes to obtain DICOM data suitable for import into clinical treatment planning systems [17, 18]. This approach is both cumbersome and limits flexibility, particularly when developing complex virtual phantoms or dynamic simulations. DICOMator addresses these critical limitations by integrating voxelisation directly within the Blender environment, enabling interactive, real-time creation and refinement of synthetic CT datasets. Its intuitive interface streamlines the entire process, allowing researchers to seamlessly generate and export phantoms, including those with complex geometries and spatially varying Hounsfield Units (HU).

Additionally, the novel integration of Blender’s built-in animation features facilitates the generation of advanced 4D CT datasets, significantly expanding the scope of phantom-based studies and enhancing the practical utility for medical physics research and quality assurance applications. This functionality is particularly valuable for simulating anatomical movements in radiotherapy treatment planning, where organ motion can significantly impact treatment accuracy and robustness. In radiation therapy, accounting for organ motion (such as respiratory motion of lung tumours) is crucial for accurate dose delivery and minimising damage to healthy tissue. By providing 4D CT capabilities, DICOMator facilitates the development and evaluation of advanced motion management strategies in a controlled, customisable environment.

The tool’s output in DICOM CT format ensures compatibility with existing radiation oncology workflows and treatment planning systems. This seamless integration allows for easy importation of synthetic CT images, including 4D datasets, into radiotherapy planning software. Such capability proves beneficial for educational purposes, research and commissioning of new treatment techniques, and the design and evaluation of novel phantoms.

**Fig. 1:**
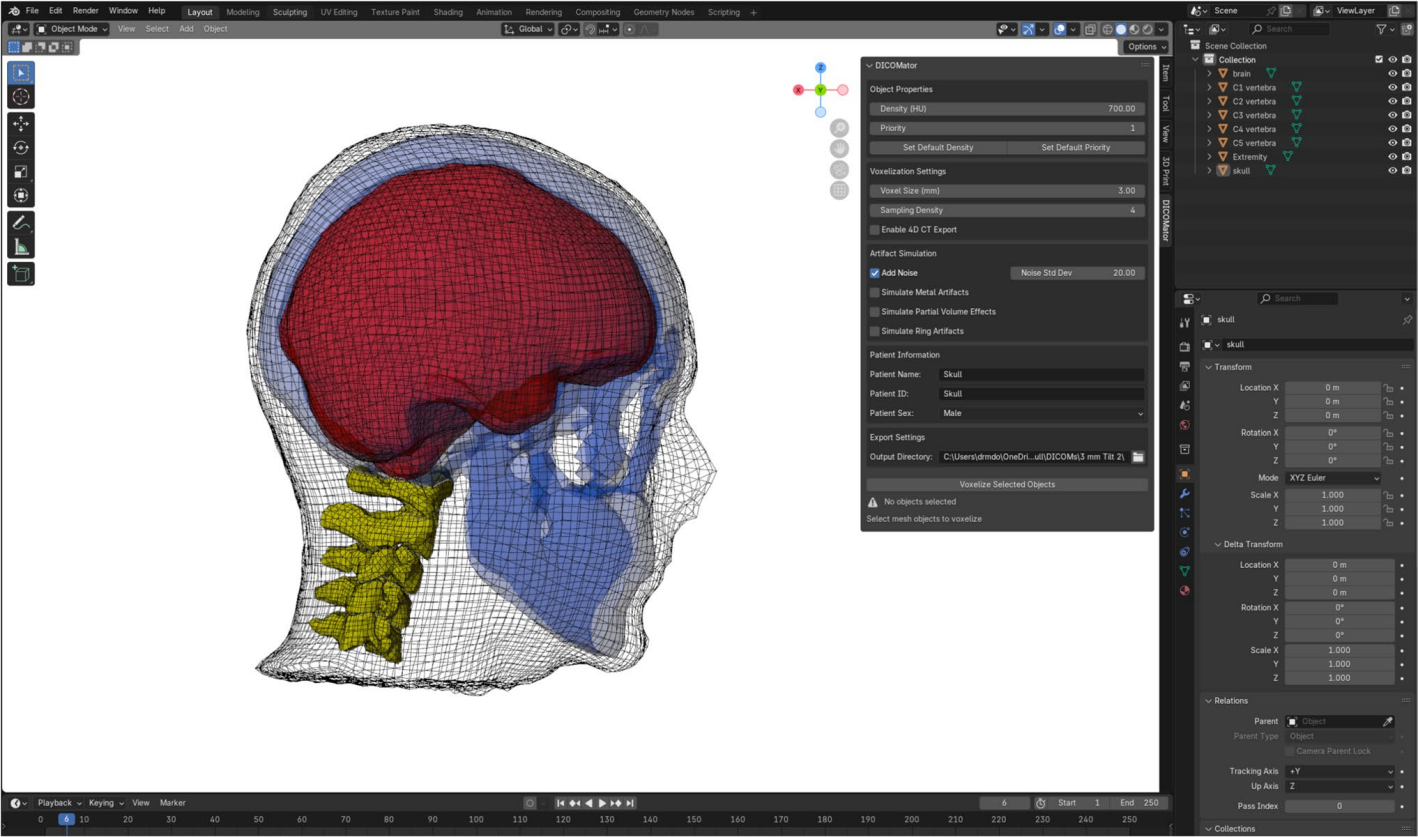
The Blender and DICOMator user interface. An example 3D model of a human head with vertebrae, brain, skull, and external volume are shown. The DICOMator panel is shown in the top right corner of the white viewport. Adjustable settings in the panel include object density (HU), overlapping structure density priority, output CT resolution (mm), artefact simulation options, patient demographic information and export location

DICOMator’s user-friendly interface (Fig. 1) within Blender [11] streamlines the workflow, offering intuitive controls for various parameters and settings. This ease of use enhances productivity when working with multiple mesh objects and animations, making it an efficient tool for creating complex, customised synthetic CT datasets.

While synthetic data offers many advantages, it is important to acknowledge potential limitations. Synthetic datasets may not fully capture the complexity and variability of real patient data, and in the case of training machine learning models, care must be taken to ensure that models trained on synthetic data perform well on real-world cases. Additionally, the creation of realistic synthetic data requires expertise in both medical imaging and 3D modelling.

This paper presents the development and implementation of DICOMator, detailing its features—including the 4D CT capabilities—underlying algorithms, and potential applications in medical physics. Optimisation strategies employed to improve performance are discussed, such as restricting voxelisation to relevant regions and efficient density assignments. The utility of DICOMator is demonstrated through three case studies: a simple lung phantom to illustrate basic functionality, a semi-realistic cranial CT scan demonstrating complex anatomical modelling and simulated tumours, and a thoracic 4D CT scan featuring multiple breathing phases to demonstrate 4D imaging capabilities and realism. These examples highlight DICOMator’s versatility in generating diverse and complex synthetic CT data suitable for various research and educational purposes, from basic quality assurance to advanced motion management studies.

## Method

DICOMator, written in Python [13] using the Blender scripting API [19], facilitates the conversion of complex 3D meshes into medical DICOM CT images. The extension not only voxelises the selected meshes but also simulates various imaging artefacts commonly encountered in clinical CT scans. The following sections provide a detailed description of the steps taken by the code and examples of how researchers can use its features.

A mesh object is a collection of vertices, edges, and faces that define the shape of a 3D object. These objects are typically created using computer-aided design (CAD) software or 3D modelling tools like Blender [11]. Mesh objects can represent complex anatomical structures, medical devices, or any other three-dimensional structures relevant to medical imaging and radiation therapy planning. The flexibility of 3D mesh objects allows for precise representation and manipulation of both simple geometries and intricate anatomical details, making them ideal starting points for generating synthetic CT datasets.

The conversion of these mesh objects into a format compatible with medical imaging systems involves a process called voxelisation. Voxelisation transforms the continuous geometry of a mesh into a discrete 3D grid of voxels, each representing a specific density value. In the case of CT medical imaging, these values are typically “Hounsfield units,” which measure the amount a photon beam is attenuated by the material in each voxel. This attenuation is related to physical or electron density of the material using a CT density conversion curve in a radiation oncology treatment planning system.

The voxelisation process converts the vector-based representation of 3D models to the raster-based format of CT images, enabling the creation of synthetic datasets that visually mimic real CT scans.

### Voxelisation process

The voxelization method implemented in this study employs an adaptive sampling technique designed to convert 3D mesh objects into a detailed voxel grid representation suitable for generating synthetic DICOM image series. The voxelisation procedure comprises the following steps:

#### Bounding box calculation

The spatial extent of the voxel grid is determined by calculating the combined bounding box of all selected mesh objects. Minimum (bbox_min_) and maximum (bbox_max_) coordinates of the bounding box are computed and stored for subsequent processing.

#### Grid dimensions calculation

The dimensions of the voxel grid are established based on the computed bounding box size. The number of voxels along each dimension is determined by dividing the bounding box dimensions by the voxel size and rounding up to ensure full coverage.

#### Voxel grid initialization

A three-dimensional numpy [20] array is initialised to represent the voxel grid, initially populated with air-equivalent HU values (− 1000 HU by default). Concurrently, a mask array of identical dimensions is initialised to track boundary voxels.

#### Mesh data preparation

Each mesh object undergoes data preparation involving retrieval of density and priority attributes, creation, and triangulation of a BMesh representation, and construction of a Bounding Volume Hierarchy (BVHTree) to facilitate efficient spatial queries in Blender. The priority attribute is specified by the user and instructs the add-on which HU value to use when there are overlapping structures. BMesh is Blender’s dynamic mesh editing system. It provides a flexible data structure for modifying vertices, edges, and faces. BVHTree is a hierarchical spatial data structure that speeds up geometric queries like ray-casting and nearest-neighbour searches, analogous to efficient collision detection in physics simulations. Prepared mesh data is stored in a structured list for subsequent voxelisation steps.

#### First pass: low-resolution sampling

**Fig. 2:**
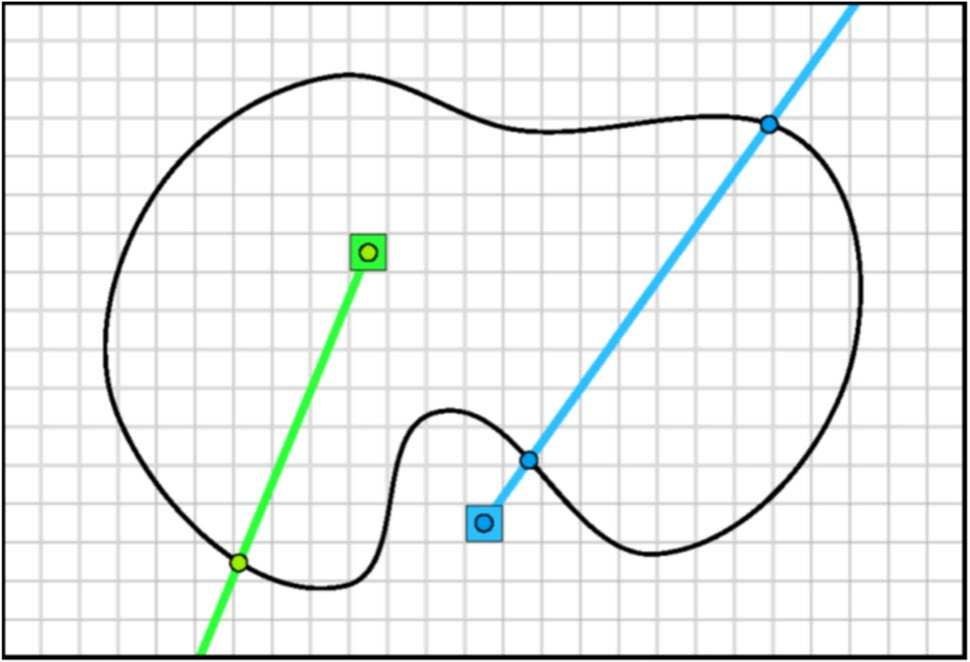
Illustration of the Ray Tracing approach used in the current work to voxelise a 3D mesh into a raster representation of the object. The green ray intersects with the exterior surface of the mesh once (and odd number of intersections) and therefore is classified as inside the mesh. The blue ray intersects twice (even) and therefore is classified as outside the mesh

An initial, low-resolution sampling pass is performed using a ray casting [21] approach to determine if sample points are inside mesh structures (Fig. 2). Sample points are generated at the geometric centre of each voxel, and neighbouring voxels are evaluated for differences in density or internal/external status to detect boundary voxels.

#### Boundary detection

The voxel grid is segmented into manageable chunks to optimise memory usage during processing. For each voxel, ray casting is applied to ascertain if the voxel centre is located within a mesh volume. Density and priority data are recorded accordingly. Differences between neighbouring voxels’ density or inclusion status are analysed to identify boundary voxels, which are marked within the boundary mask for further processing.

#### Second pass: high-resolution sampling

Boundary voxels identified during the initial sampling pass undergo a secondary, higher-resolution sampling procedure. Within each boundary voxel, multiple high-resolution sample points are generated, and a ray casting approach is again employed to determine if each sample point lies within a structure.

The finalised voxel grid, with density values precisely assigned through adaptive sampling, is then stored. This adaptive voxelisation approach effectively balances computational efficiency with high spatial accuracy, providing detailed structure representation particularly suited to medical imaging applications.

### Artefact simulation tools

After iterating over all voxels within the bounding box and processing all selected 3D mesh objects, the code produces an idealised CT dataset without artefacts. To enhance the realism of this synthetic dataset, the software offers several options to simulate typical image artefacts commonly found in CT scans.

#### Noise

Users can add Gaussian noise to replicate the random noise inherent in CT imaging systems. The add_noise function simulates random noise in the synthetic CT image by adding Gaussian noise to the voxel grid representing the image data. Specifically, if the noise_std_dev (standard deviation of the noise) parameter is greater than zero, the function generates a noise array of the same shape as the voxel grid, with values drawn from a normal distribution centred at zero. This noise array is then added to the original voxel grid, introducing random fluctuations in voxel intensities throughout the entire 3D image. This process approximates the random noise commonly present in real CT images due to factors like electronic sensor noise and photon counting statistics, thereby enhancing the realism of the simulated data for testing and validation purposes.

#### Metal artifacts

The metal artefact simulation replicates the artefacts caused by metal implants, appearing as streaks emanating from high-density regions. The function simulates bright radial streak artefacts in a computed tomography (CT) image by altering voxel values around high-density regions within each axial slice. It operates by iterating over each slice along the axial direction of the 3D voxel grid representing the CT image. For every slice, it identifies voxels that exceed a specified metal density threshold, classifying them as metal voxels.

Around each identified metal voxel, the function projects multiple rays outward in various directions determined by the num_angles parameter, which specifies the number of angles to cover a full 360-degree circle. Along these rays, it increases the intensity of the voxel values to create bright streaks. An attenuation factor based on the radial distance from the metal voxel is applied, causing the intensity effect to diminish as it moves away from the source, mimicking the physical attenuation observed in real CT images.

After modifying the voxel intensities along all rays for all metal voxels in the slice, the function ensures that the voxel values remain within the valid Hounsfield Unit (HU) range for CT images by clipping them appropriately. It then updates the original voxel grid by replacing the original slice with the modified slice containing the simulated artefacts.

By repeating this process for each slice containing metal voxels, the function effectively simulates bright radial streak artefacts commonly observed around metal objects in CT images.

#### Partial volume effects

Partial volume effects occur in medical imaging when a single voxel contains multiple tissue types, resulting in an averaged HU intensity for that voxel. This phenomenon leads to blurring of boundaries between different tissues and can affect the accuracy of structure delineation, especially for small structures or regions with rapid density changes.

The apply_partial_volume_effect function simulates the partial volume effect in computed tomography (CT) images by applying a Gaussian blur to the voxel grid representing the image data. It uses the gaussian_filter function from the scipy ndimage[22] module, where the sigma parameter determines the standard deviation of the Gaussian kernel used for blurring. By convolving the voxel grid with this Gaussian kernel, the function smooths the image and mimics the averaging of HU that occurs when a single voxel includes multiple tissue types in real CT imaging.

#### Ring artefacts

The ring artefact simulation mimics simple ring artefacts that can occur due to detector imperfections or calibration errors in CT scanners. The simulate_ring_artefacts function simulates ring artefacts in the synthetic computed tomography (CT) image by introducing concentric sinusoidal ring patterns to each axial slice of the voxel grid. For each slice, it calculates the radial distance from the centre of the slice to every voxel using the Euclidean distance. It then generates a ring pattern by applying a sine function over these radial distances, with the frequency parameter controlling the number of rings. The resulting ring pattern is scaled by the specified intensity and added to the original slice data, modifying the voxel intensities to mimic ring artefacts. This process is repeated for all slices in the voxel grid, introducing ring artefacts throughout the entire CT volume.

### DICOM CT series export

Once all post-processing effects have been applied, the voxel grid is exported as a series of DICOM CT image slices, compatible with medical imaging software. The code uses the “pydicom” [23] library to create and populate DICOM file datasets, generating unique identifiers (UIDs) for the study, series, and instances using the “pydicom.uid.generate_uid” function. Essential metadata such as patient information, study and series dates, modality-specific attributes, and image type are assigned to each DICOM file. The code sets modality-specific attributes such as Modality (CT), Manufacturer and Software Versions (1.0), and assigns patient information, which can be customised.

Spatial information of each slice is represented using the Image Position (Patient) and Image Orientation (Patient) tags, calculated based on the user defined voxel size and bounding box coordinates.

For 4D datasets, temporal position identifiers are added to the DICOM files to represent different temporal phases. The code includes temporal identifiers such as the temporal_position_index and NumberOfTemporalPositions tags, allowing the DICOM series to represent time-varying data.

**Fig. 3:**
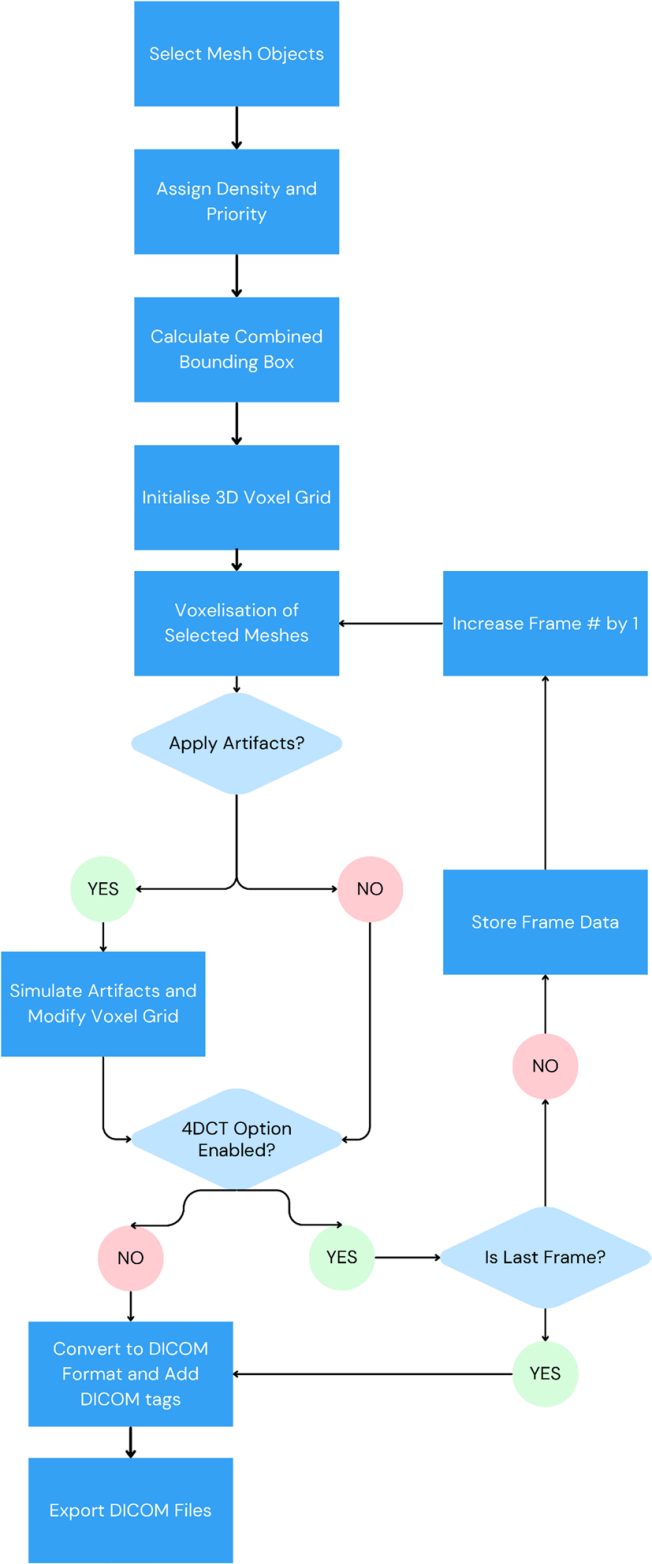
The workflow of the code to convert a collection of 3D mesh objects in Blender into a voxelised DICOM CT series

A flowchart representing this workflow is shown in Fig. 3 and a list of all DICOM tags exported in the Synthetic DICOM series can be found in the code repository [10].

### Testing and verification

To demonstrate the capabilities of the add-on, three synthetic CT datasets of varying complexity were created. Each example was designed to illustrate a potential use case for medical physics research.

#### Simple lung phantom using basic 3D shapes

A simple lung phantom was created, consisting of three cylindrical meshes, stretched in the x–y (lateral, ant-post) plane to simulate the appearance of a basic lung anatomy. One cylinder represented the external contour of a human body, while the other two cylinders represented the left and right lung volumes (supplementary Fig. 1).

The purpose of this example was to demonstrate DICOMator’s ability to generating simple phantoms for QA and commissioning in treatment planning systems. The example also served as a demonstration of the tools usefulness for testing deformable image registration algorithms and simulating metal artefacts.

The external cylinder was assigned a Hounsfield Unit (HU) value of 0, to approximate that of water. The two lung cylinders were assigned a value of -200 HU, approximating the density of a typical lung material.

Two different synthetic DICOM CT image series were created from this geometry; one with the lung volumes located 1 cm superior to the centre of the external structure and a second with the lung volumes positioned 1 cm inferior. This was done to demonstrate the potential of this software for validating rigid and deformable image registration (DIR) algorithms. The two CT datasets were exported as DICOM series and then imported into Raystation (Raysearch Laboratories, Sweden) Version 12A where the external and lung volumes were automatically segmented using threshold-based segmentation.

The two CT volumes were then deformably registered to each other using the Hybrid intensity and structure-based algorithm. The default deformation strategy was selected in Raystation using the correlation coefficient as the similarity measure. The lung volumes were selected as the controlling ROIs.

Once registered, twelve points were placed on the superior and inferior edges of the lung volumes to determine the magnitude of the displacement field between the two datasets to confirm that the value matched the 2 cm value originally simulated in Blender.

In addition, as a demonstration of the metal artefact simulator tool, a third CT dataset, using the simple lung phantom, was exported from Blender, where a small spherical volume was placed in the region between the lung volumes and assigned a HU value of 2000, representative of a high-density implant.

#### Synthetic cranial CT scan

For the second example, a pseudo-realistic human cranial CT was created using a multi-step process. The purpose of this example was to demonstrate the ability of the add-on to produce synthetic CT volumes with simulated tumours for dose calculations in treatment planning systems. It also served to demonstrate a more complex example of CT image registration applications.

First, a CT image of a human head was obtained from the CERMEP-IDB-MRXFDG dataset [24]. The CT image series was imported into Slicer 3D [25] where the Total Segmentator [26] deep learning extension was used to automatically contour the patient’s external, brain, vertebrae, and skull volumes.

**Fig. 4:**
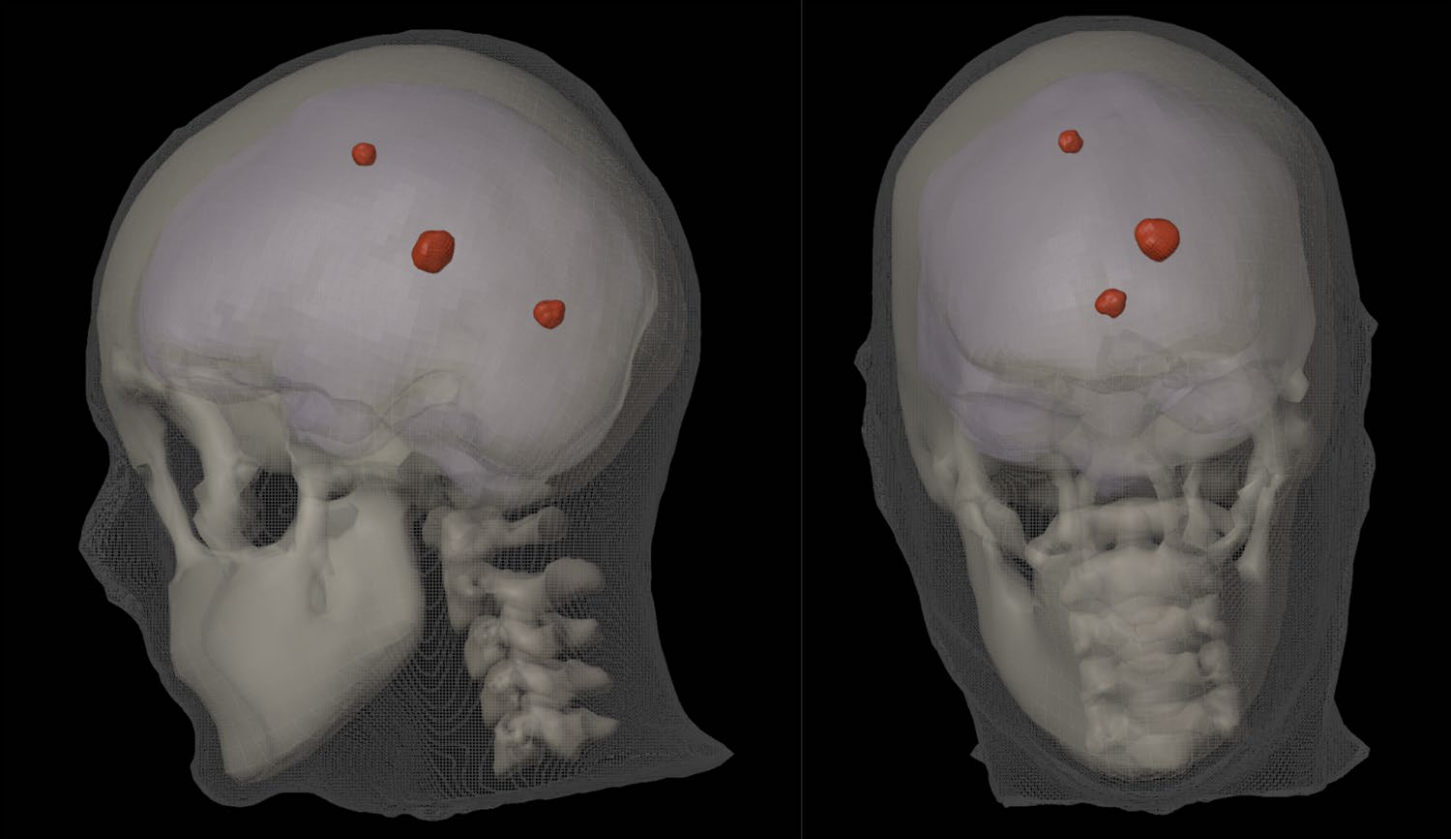
The external, skull, vertebrae and brain segmentations produced by Total Segmentator. Additionally, three simulated brain lesions were added using Blender’s sculpting tools (red)

After segmentation, these volumes were converted to 3D models and exported as.obj files, a common text-based 3D format. The resulting models, representing the external, skull, vertebrae, and brain structures, were then imported into Blender (Fig. 1). DICOMator was used to assign an appropriate Hounsfield Unit (HU) value to each structure: 0 HU for the external structure, 1000 HU for the skull, 1000 for the vertebrae and 200 HU for the brain. Three simulated lesions were created using spherical meshes and Blender’s sculpting tools (Fig. 4). Each lesion was assigned a HU value of 250 HU. The noise artefact tool was enabled with a Gaussian noise sigma of 5 HU.

One additional synthetic CT was generated with identical geometry to the first except a pitch of 3° was applied to the skull, external and brain volumes relative to the vertebrae to simulate a head tilt. Both geometries were voxelised with a resolution of 2 mm and exported as DICOM CT series to the Raystation treatment planning system.

A simple VMAT plan was created to deliver a uniform dose of 2 Gy to the three simulated PTV targets while minimising dose to the brain volume. This was achieved using the dose falloff objective in the optimiser.

The additional synthetic CT scan with 3° tilt was imported into Raystation and rigidly registered to the first using the default settings. The rotational values reported by the rigid registration were compared with the expected pitch values applied in Blender.

#### Thoracic 4D CT scan with realistic anatomy

Finally, the 4D CT feature of DICOMator was demonstrated by creating a 4D thoracic DICOM CT series with ten temporal phases. This example also served to demonstrate the levels of complexity and realism achievable with the DICOMator tool.

A thoracic CT scan was obtained from the Visible Human Project dataset [27]. The dataset was subsequently imported into Slicer 3D [25], where the Total Segmentator [26] extension was used to automatically segment the external contour of the patient, the lung volumes, and the heart.

**Fig. 5:**
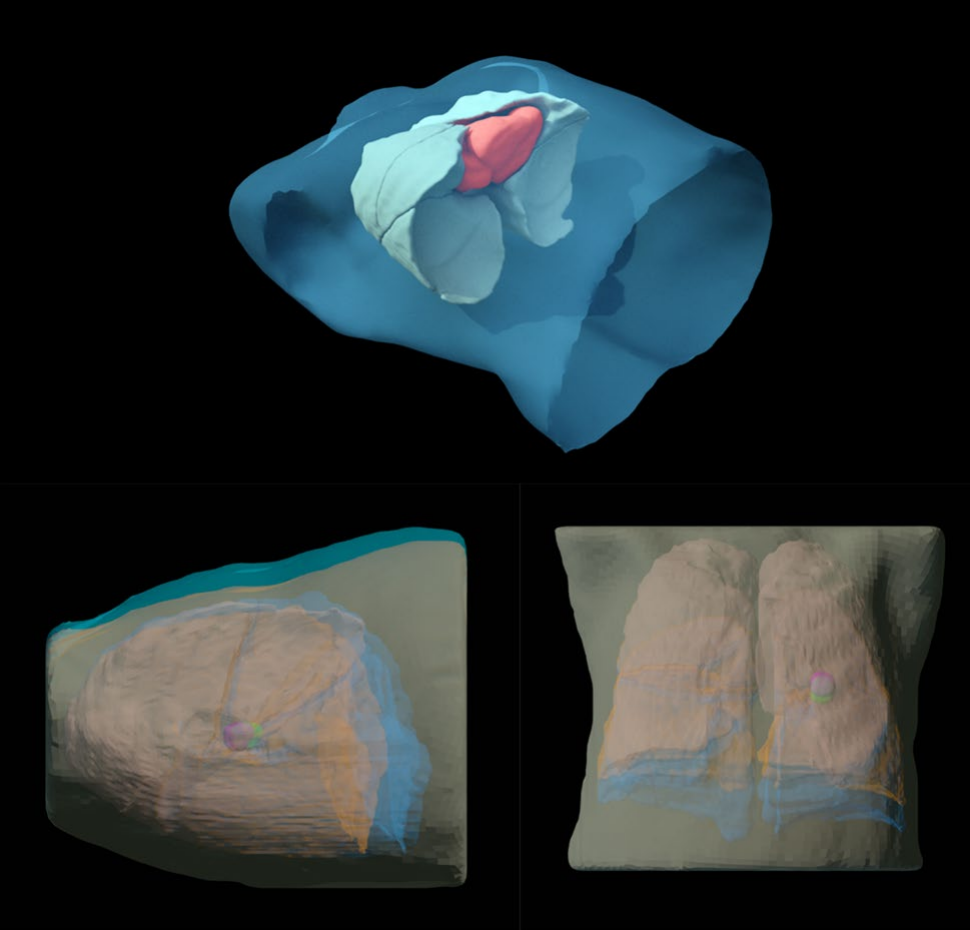
Top—Visualisation of the 3D lung geometry in Blender. Dark blue: External, Light blue: Lungs, Red: Heart. Bottom—Comparison of the maximum inhalation (blue) and exhalation phases (orange) of the animated breathing cycle. The diaphragm and external contour show the largest anatomical changes

These segmentations were then exported in OBJ format from Slicer3D and imported into Blender. Within Blender, the external structure, lungs, and heart regions were assigned Hounsfield Unit (HU) values of 0, − 200, and 100, respectively, to simulate typical tissue densities for each region (Fig. 5). The noise artefact option was enabled with a Gaussian noise sigma of 5 HU.

To simulate the respiratory motion of the patient, the lung and external volumes were animated in Blender by performing a non-linear scaling of the 3D meshes along the superior-inferior and anterior–posterior axis. The contours were deformed in Blender using a lattice-based deformation which enabled the respiratory motion of the contours to be approximated (Fig. 5). The animation was key framed at multiple time points in the breathing cycle to produce a cyclic animation. The animation was configured to run for ten frames, representing ten breathing phases of a 4D CT.

To demonstrate the quantitate and qualitative accuracy of the synthetic datasets generated by DICOMator, a second, more complex phantom was generated from the same thoracic CT but included significantly more contoured anatomical regions generated using Total Segmentator. Additional structures included spine, ribs, liver, and stomach.

The animated 4D phantom and static high-quality phantom were voxelised with a resolution of 2.5 mm and exported in DICOM format to the Raystation treatment planning system. Additionally, the original patient CT from the Visible Human Project used to generate these synthetic datasets was imported into Raystation where it was co-registered and compared with the synthetic CT dataset. The synthetic and original datasets were compared using the image fusion tools in Raystation to highlight the potential realism of the synthetic datasets achievable with DICOMator and, identify differences and similarities compared with the original patient CT.

## Results

### Simple lung phantom using basic 3D shapes

**Fig. 6:**
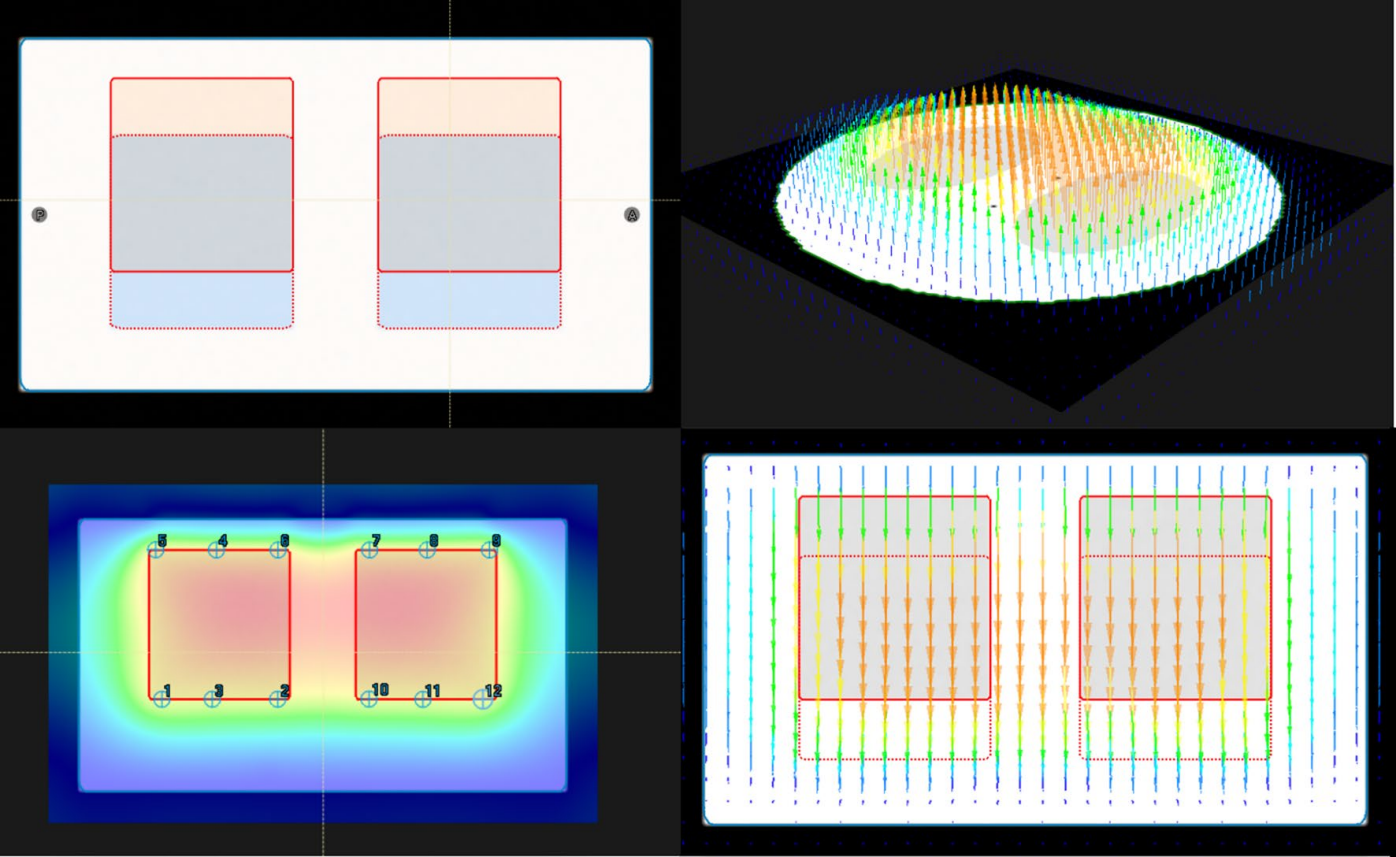
Demonstration of the proposed workflow for validating deformable image registration algorithms. Top Left: The difference map between the first and second lung phantom CT illustrating the simulated lung motion in the superior-inferior direction. Top Right: An axial slice of the lung phantom with a 3D vector displacement map corresponding to that slice. Bottom Left: The displacement intensity map of the lung CT. Twelve points are shown which were used to calculate the magnitude of the displacement. Bottom Right: The original (solid red) and deformed (dotted red) contours of the lung volumes with vector displacement field overlaid

The two synthetic lung phantom CTs were deformably registered to each other in Raystation, with the displacement field visualised and quantified. Twelve points were placed at the superior and inferior edges of the lung volumes and the magnitude of the displacement field between the deformably registered CT images was quantified (Fig. 6). The magnitude of the displacements in the R-L, I-S and P-A directions for the twelve points were 0.001 ± 0.007, 1.998 ± 0.055, and 0.001 ± 0.035 cm, respectively. This result matches the expected displacement of 0, 2 and 0 cm simulated in Blender.

In addition, a visual demonstration of the metal artefact simulation tool was performed. The result of this is illustrated in supplementary Fig. 2 where a series of streaks emanating from the simulated metal object can be seen. The radial attenuation, number of streaks and intensity of the streaks are all customisable via the DICOMator user interface.

### Synthetic cranial CT scan

The synthetic cranial CTs generated in Blender were exported as a DICOM CT series and successfully imported into the Raystation treatment planning system. The synthetic CT image series with simulated HU values was converted to physical density using the clinical HU-to-Density conversion curve in the Raystation. The average physical densities for the normal tissue (external—Brain—skull), brain and skull regions reported by Raystation were 1.003, 1.070 and 1.66 g/cm^3^, respectively.

**Fig. 7:**
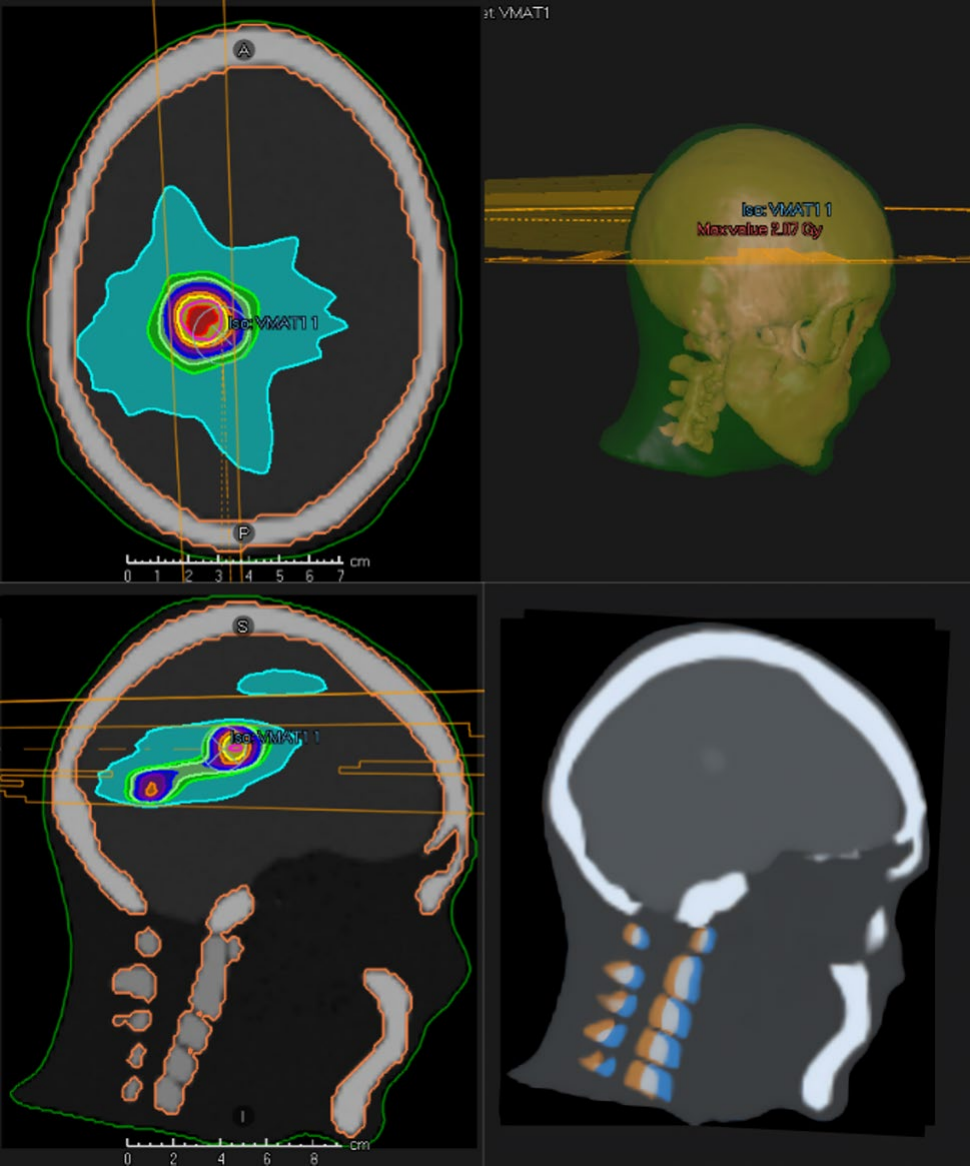
Left—Visualisation of the calculated dose from Raystation TPS on the synthetic cranial CT image for a simulated single isocentre, three target VMAT treatment. Top Right—External and skull volumes and VMAT arc trajectories shown in Raystation. Bottom Right—Fusion between the first synthetic CT and the 3° pitch simulated in Blender. One of the simulated lesions can be seen in the mid-brain volume

To demonstrate a potential application of this synthetic dataset, a sample Volumetric Modulated Arc Therapy (VMAT) radiotherapy treatment plan was created in Raystation. This plan consisted of two full VMAT arcs designed to uniformly irradiate the three synthetic tumours generated in Blender, while minimising dose to the surrounding brain tissue using the dose fall-off objective (Fig. 7). This example highlights the potential of the add-on for generating datasets with simulated lesions for treatment planning and dosimetry studies.

The additional synthetic CT scan with the 3° pitch was rigidly registered in Raystation. The rotational offset between the two datasets required to align the skull volumes reported by Raystation was 2.9° pitch (Fig. 7). This level of agreement successfully demonstrates the usefulness of the DICOMator add-on to assist in the validation of image registration algorithms.

### Thoracic 4D CT scan with realistic anatomy

**Fig. 8:**
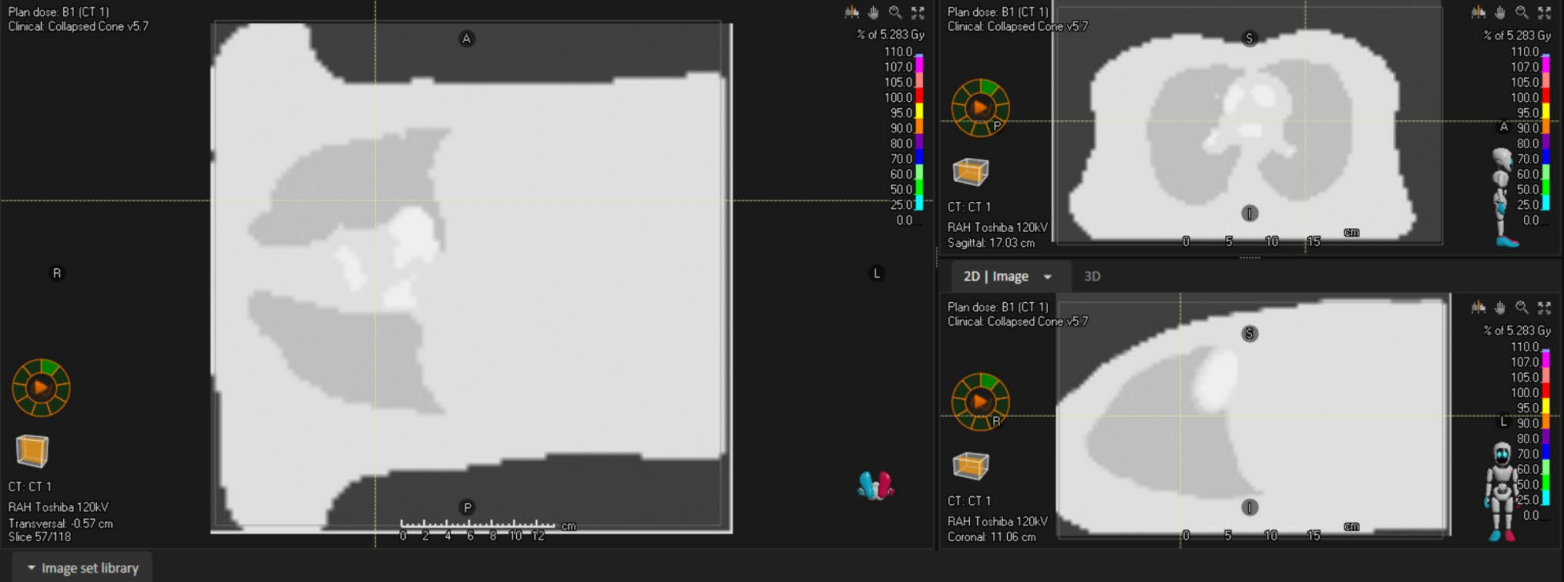
The 4D CT lung dataset in the Raystation treatment planning system

Raystation was able to successfully import and interpret the synthetic lung dataset as a 4D CT volume and display the animated lung volume (Fig. 8). Raystation was also able to successfully recognise and use this dataset in the robust optimisation and robust evaluation tools.

**Fig. 9:**
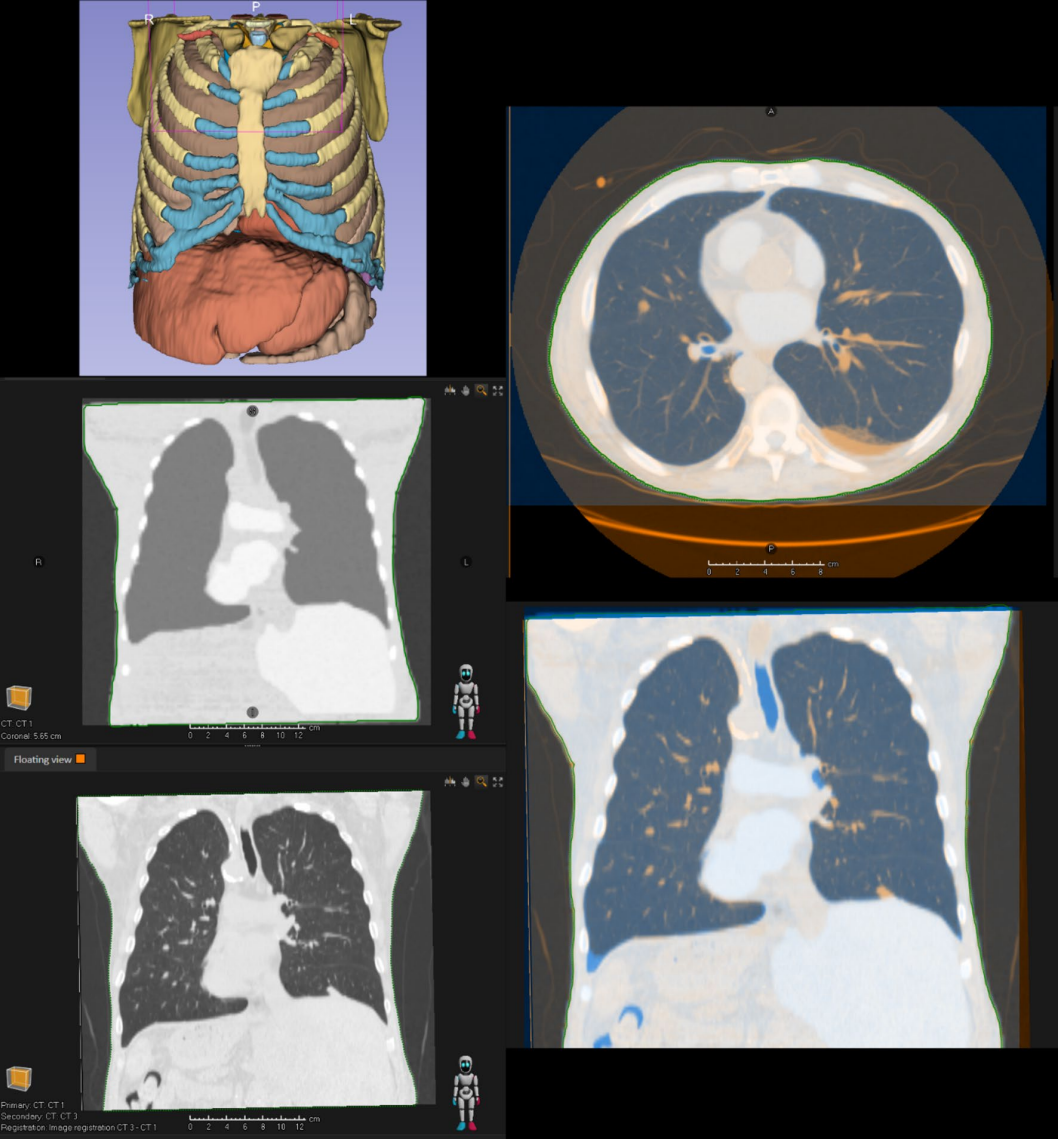
Comparison of the original patient CT and the synthetic dataset generated using DICOMator. Top left—The 3D anatomical model used to generate the synthetic dataset. Middle Left—The synthetic CT dataset. Bottom Left—The original “ground-truth” patient CT dataset. Right—The image fusion between the two datasets as shown in Raystation

When the second, more detailed, synthetic lung CT was compared directly with the original patient CT, a high degree of similarity was observed, shown in Fig. 9. Most notably, the anatomical features of both datasets were geometrically similar. Differences in HU values between the two datasets were most apparent due to DICOMator’s limitation of only generating homogeneous densities for each region. Furthermore, the bronchi are visible in the lungs of the original patient CT but not the synthetic dataset. This is due to the bronchi not being automatically contoured on the original CT and therefore not being considered in conversion to the synthetic equivalent dataset.

These results demonstrate the capability of the DICOMator add-on to generate qualitatively realistic 4D CT datasets, which can be valuable for research and clinical applications in respiratory motion management and adaptive radiotherapy.

## Discussion

The work presented herein demonstrates a wide range of potential applications for the proposed DICOMator add-on in the field of medical physics research and development. For image processing, it excels as a tool for validating various image registration algorithms, including rigid and deformable registration techniques. Researchers can create synthetic datasets with pre-defined deformations, providing a controlled environment with known ground truth for assessing the accuracy and robustness of new algorithms.

Furthermore, the effectiveness of the add-on in creating simple or realistic phantoms for various medical physics applications has been demonstrated. Notably, the software simulates various CT artefacts, including partial volume effects, metal artefacts, and noise. These artefacts, which are common in clinical CT scans, add a layer of realism to the synthetic dataset, making it particularly valuable for research and educational purposes.

In this study, two open-source patient datasets were employed as a template for the generation of the initial synthetic phantoms. While this approach may appear counterintuitive, given that the primary objective of the DICOMator tool is to produce fully synthetic datasets, it serves a critical role in demonstrating the tool’s flexibility. Following segmentation, the anatomical structures derived from the patient CT data were converted into individual 3D meshes, which could then be programmatically manipulated. This facilitated the generation of a wide range of synthetic phantoms through systematic variation of parameters such as tissue density, organ size, and respiratory motion as well as the addition of synthetic tumours not present on the original patient CT. As a result, a variety of synthetic datasets can be derived from a single anatomical template. Thus, the initial use of real patient data does not compromise the synthetic nature or purpose of the DICOMator framework; rather, it underscores its utility and adaptability for diverse applications in medical physics research.

For machine learning applications, the add-on may prove invaluable for generating synthetic data for model training. This is particularly beneficial when real patient data is scarce or when simulating specific rare conditions. Initially, machine learning models can be trained using synthetic data generated by DICOMator and subsequently fine-tuned with a smaller sample of real patient data. Moreover, using synthetic data addresses ethical concerns related to patient privacy and data protection.

In education, DICOMator enables the creation of customised datasets for teaching and training. For instance, educators can design cases that demonstrate the effects of metal artefacts on dose calculations or simulate various stages of tumour growth, providing students with hands-on experience across a diverse range of scenarios without using real patient data.

For treatment planning and quality assurance, DICOMator offers several key applications. It enables the verification of dose calculation accuracy in the presence of metal artefacts, a common challenge in radiotherapy planning. By creating controlled scenarios with known artefact patterns, medical physicists can assess the impact of these artefacts on dose calculations and develop strategies to mitigate their effects. Furthermore, researchers can use the tool to study the effect of image noise on dose distributions, helping to optimise imaging protocols and understand the relationship between image quality and treatment planning accuracy.

In motion management, DICOMator’s capability for simulated organ motion allows for the development and testing of advanced motion management strategies in radiation therapy. This feature is particularly relevant for improving the precision of treatments in areas affected by respiratory or cardiac motion. By creating realistic 4D datasets, researchers can evaluate different motion compensation techniques and develop more effective strategies for delivering accurate treatments to moving targets.

DICOMator also plays a role in quality assurance by facilitating the creation of “ideal” physics phantom datasets. This ensures consistent quality across different institutions and helps maintain high standards in medical physics practices. DICOMator offers flexibility in creating custom phantoms by integrating seamlessly with the Blender environment, making it uniquely positioned for research and educational purposes.

Looking ahead, DICOMator opens new research directions, such as developing advanced artefact reduction techniques for 4D imaging and optimising protocols for adaptive radiotherapy.

### Limitations

Despite DICOMator’s significant capabilities, it is important to acknowledge several limitations. The current version does not accurately replicate all types of CT artefacts, particularly more complex ones such as scatter or beam hardening from highly attenuating materials. Nor does it generate artefacts based on realistic physics models. In addition, the current version of the software assigns uniform HU values for each structure, which, while improving over existing tools [17, 18], may not fully capture the heterogeneity of real tissues. While noise can be added, it may not perfectly mimic the complex noise patterns found in real CT scanners. The primary aim of this work was to develop synthetic datasets which were qualitatively similar to real CT data for specific use cases. Further work is needed to enhance the quantitative accuracy of the synthetic images.

Another limitation relates to voxelisation time. Complex meshes with intricate topology require a larger number of sample rays to correctly voxelise the geometry, with the voxelisation time increasing with the selected number of rays. All examples in this study were generated on a PC with a 13th Generation Intel (Santa Clara, California, USA) Core® i9-13900HX CPU with 32 Gb of RAM. Simple phantoms, such as the lung phantom in example one, took only a few seconds to create. However, for more complex geometries, like the synthetic 4D lung in example three, the voxelisation process took approximately 30 min. The original 3D models for the external body, heart, and lungs contained between 10,000 and 15,000 vertices each. While the current code uses only the CPU, future improvements could potentially leverage GPU processing power for faster computation.

### Future work

Building upon the current capabilities of DICOMator, several avenues for future development and enhancement have been identified. The realism of the existing artefact simulations, particularly for complex artefacts such as beam hardening and scatter, could be improved. Furthermore, the add-on could be expanded to enable users to select imaging system parameters such as tube kV and mA to fine-tune the generated synthetic CT images. This would involve implementing more sophisticated models to accurately mimic realistic CT properties and artefacts, providing a more comprehensive tool for researchers and clinicians studying image quality and artefact reduction techniques.

Extending DICOMator’s capabilities to simulate other imaging modalities, such as MRI, PET, and SPECT, would expand the tool’s utility in multi-modal imaging research. This would involve developing new algorithms to mimic the unique characteristics and artifacts of each modality. Furthermore, implementing more sophisticated tissue models that better represent the heterogeneity and complexity of human anatomy could enhance the tool’s realism. This could include incorporating texture maps for organs and simulating variations in tissue density within structures.

To improve performance, optimising the voxelisation process by leveraging GPU computing could significantly reduce processing times for complex geometries and enable the creation of higher-resolution synthetic datasets. Integration with popular deep learning frameworks through plugins or APIs would facilitate the generation of large-scale synthetic datasets for training and validation of AI models in medical imaging.

Enhancing the 4D simulation features to include more complex motion patterns and physiological processes, such as cardiac motion, blood flow, and contrast agent dynamics, would further expand DICOMator’s capabilities in dynamic imaging simulation.

These future developments aim to further establish DICOMator as a comprehensive, versatile tool for medical imaging research, education, and quality assurance, addressing the evolving needs of the medical physics community.

## Conclusion

In summary, DICOMator has been demonstrated to be useful in medical physics research, offering a flexible and versatile solution for generating synthetic CT datasets. This open-source Blender add-on bridges a crucial gap in research, education, and quality assurance by enabling the creation of customisable, realistic CT images without relying on actual patient data or physical phantoms. Its open-source nature fosters collaborative improvement and widespread adoption within the medical physics community.

Demonstrated through examples such as simple lung phantoms, pseudo-realistic human head CTs, and 4D CT lung scans, DICOMator enhances various aspects of medical physics research, including validating image registration algorithms, training machine learning models, and improving treatment planning and motion management strategies.

While acknowledging current limitations, such as the simplification of certain tissue properties and artifacts, DICOMator provides a solid foundation for future developments.

As medical physics continues to evolve, tools like DICOMator will play an increasingly crucial role in driving innovation, enhancing educational practices, and improving patient care. By offering researchers, educators, and clinicians a flexible platform for synthetic CT generation, DICOMator paves the way for more robust, efficient, and ethical advancements in medical imaging and radiation therapy, enhancing treatment planning accuracy and patient outcomes in radiation oncology.

## Supplementary Information

Below is the link to the electronic supplementary material.

Supplementary file1 (DOCX 186 KB)
