# Supplementary material for: An open-source tool for converting 3D mesh volumes into synthetic DICOM CT images for medical physics research
Source: Phys Eng Sci Med. 2025 Jul 24;48(4):1525–38. doi: 10.1007/s13246-025-01599-x (PMC12738608; doi:10.1007/s13246-025-01599-x)
Supplement: Supplementary file 1 — Supplementary file1 (DOCX 186 KB) [file 13246_2025_1599_MOESM1_ESM.docx]

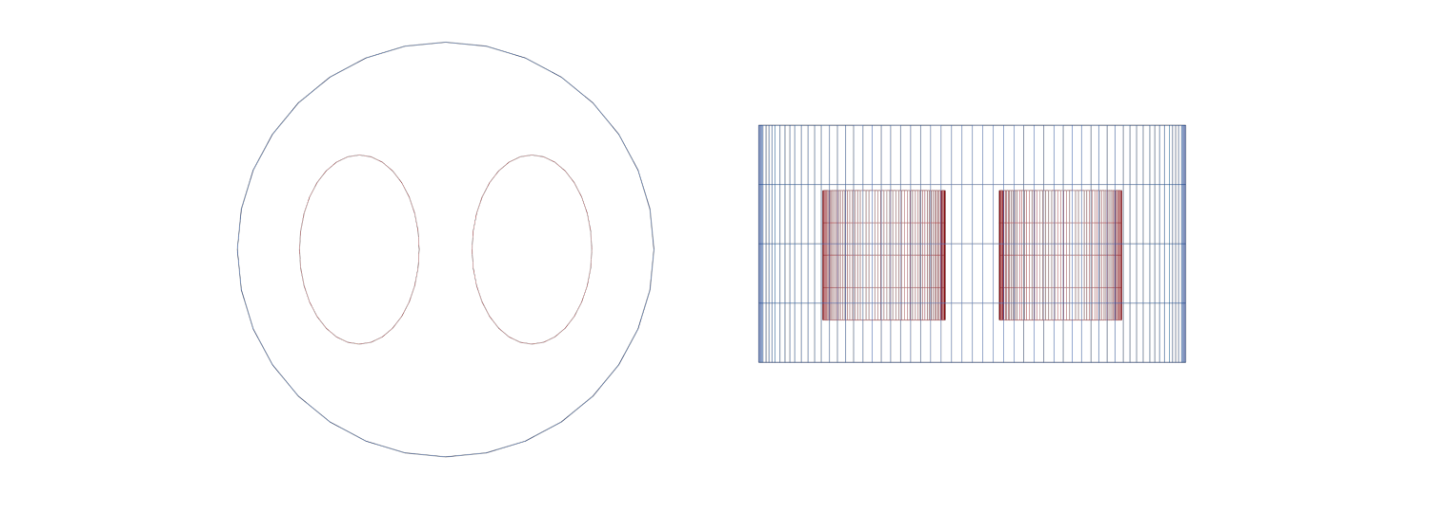


Supplementary Figure 1. Visualisation of the simple lung phantom geometry. Left: axial view showing the external body contour in blue and lung volumes in red and Right: Coronal view.


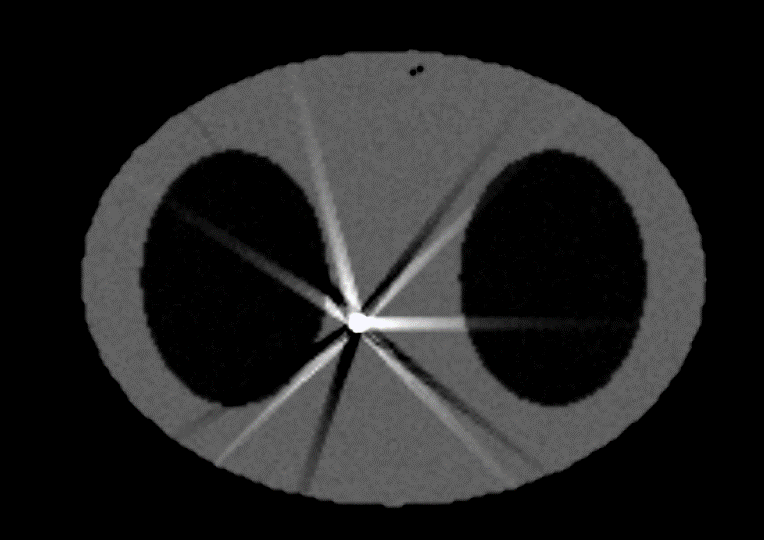


Supplementary Figure 2. A visual demonstration of the metal artefact simulator implemented in the current work. The 2000 HU spherical volume between the lung volumes is creating a simulated streaking artefact.
